# Structural changes in left fusiform areas and associated fiber connections in children with abacus training: evidence from morphometry and tractography

**DOI:** 10.3389/fnhum.2013.00335

**Published:** 2013-07-04

**Authors:** Yongxin Li, Yunqi Wang, Yuzheng Hu, Yurong Liang, Feiyan Chen

**Affiliations:** ^1^Bio-X Laboratory, Department of Physics, Zhejiang UniversityHangzhou, P. R. China; ^2^School of International Studies, Zhejiang UniversityHangzhou, P. R. China; ^3^Department of Psychology and Behavioral Sciences, Zhejiang UniversityHangzhou, P. R. China; ^4^Heilongjiang Abacus AssociationHaerbin, P. R. China

**Keywords:** abacus training, fusiform gyrus, voxel-based morphometry, fiber tracking, inverse effect, children

## Abstract

Evidence supports the notion that the fusiform gyrus (FG), as an integral part of the ventral occipitotemporal junction, is involved widely in cognitive processes as perceiving faces, objects, places or words, and this region also might represent the visual form of an abacus in the abacus-based mental calculation process. The current study uses a combined voxel-based morphometry (VBM) and diffusion tensor imaging (DTI) analysis to test whether long-term abacus training could induce structural changes in the left FG and in the white matter (WM) tracts distribution connecting with this region in school children. We found that, abacus-trained children exhibited significant smaller gray matter (GM) volume than controls in the left FG. And the connectivity mapping identified left forceps major as a key pathway connecting left FG with other brain areas in the trained group, but not in the controls. Furthermore, mean fractional anisotropy (FA) values within left forceps major were significantly increased in the trained group. Interestingly, a significant negative correlation was found in the trained group between the GM volume in left FG and the mean FA value in left forceps major, suggesting an inverse effect of the reported GM and WM structural changes. In the control group, a positive correlation between left FG GM volume and tract FA was found as well. This analysis visualized the group level differences in GM volume, FA and fiber tract between the abacus-trained children and the controls, and provided the first evidence that GM volume change in the left FG is intimately linked with the micro-structural properties of the left forceps major tracts. The present results demonstrate the structural changes in the left FG from the intracortical GM to the subcortical WM regions and provide insights into the neural mechanism of structural plasticity induced by abacus training.

## Introduction

The fusiform gyrus (FG) is an integral part of the ventral occipitotemporal junction, a region involved widely in such cognitive processes as perceiving faces, objects, places or words (Kanwisher et al., 1997; Cohen and Dehaene, 2004; Roth et al., 2006; Mei et al., 2010; Woodhead et al., 2011). A dissociation was observed between visual processing in the left and right FG (Woodhead et al., 2011). Previous studies have implicated a particularly important role of the right FG in face perception and recognition (Kanwisher et al., 1997; Bilalić et al., 2011). The left posterior FG was labeled as the visual word form areas (Cohen and Dehaene, 2004). Spaced learning could enhance the memory by enhancing the activity of left FG (Xue et al., 2010). Specifically, these previous findings focused on the differential contribution of left and right FG, but emerging literatures are beginning to highlight the role of FG in the abacus training.

Abacus, a sort of traditional calculator, is used in China and other East Asian countries currently. It uses the position of beads to represent numbers and consists a set of rules for arithmetic operations. Abacus experts can perform arithmetic calculations of large numbers with unusual speed and accuracy. Interestingly, they can manipulate the beads skillfully not only on a physical abacus but also via an imaged abacus (Stigler, 1984; Hatta and Miyazaki, 1989; Frank and Barner, 2012). Abacus mental calculation involve many aspects of high level cognition functions including number recognition, retrieval of arithmetic facts, temporary storage of intermediate results, and manipulation of mental representations (Hanakawa et al., 2003; Chen et al., 2006). Previous neuroimaging research findings consistently revealed that abacus-trained subjects are more dependent on the fronto-parietal network in mental calculations (Hanakawa et al., 2003; Chen et al., 2006; Tanaka et al., 2012). Another important observation of the abacus training studies were that abacus experts showed an enhanced activity in the left FG during mental arithmetic (Hanakawa et al., 2003; Chen et al., 2006). A number size effect in left FG was also observed only in the abacus experts in the study by Hanakawa et al. (2003), a region hypothesized to visually represent the abacus in abacus-based mental calculations. Our recent study on the effect of abacus training on structural connectivity indicated that long-term abacus training enhanced the white matter (WM) fractional anisotropy (FA) in left occipitotemporal junction (Hu et al., 2011), a key pathway linking the ventral stream (lingual, fusiform and the parahippocampal gyrus) and the dorsal visual stream (inferior and superior parietal lobule) (Rykhlevskaia et al., 2009). Although the activation in left FG was found to be specific to the long-term abacus-trained subjects during mental arithmetic, it remains unclear whether the long-term abacus training can also lead to structural plastic changes in this region and in the fiber pathway connecting this region.

During the past decade, many findings confirmed the notion that experience and learning a particular skill could cause functional and structural reorganization of the brain (Kelly and Garavan, 2005; Johansen-Berg, 2007; May, 2011; Ansari, 2012). In the present study, we focused on the left FG. Based on previous neuroimaging findings (Hanakawa et al., 2003; Chen et al., 2006), we hypothesized that regional gray matter (GM) structures in the left FG should be affected by the abacus training. Furthermore, we expected a training-induced WM integrity increase in the fiber tracts pathway connecting left FG (Hu et al., 2011).

We combined evidences from morphometry and tractography to test our hypothesis. First, we applied an optimized method of voxel-based morphometry (VBM) on the T1-weight images (Ashburner and Friston, 2000; Good et al., 2001) to examine whether long-term abacus training may induce morphological change in the left FG. Second, we examined the changes in WM which, like GM, also appears to be susceptible to such training effect (Johansen-Berg, 2012). Diffusion tensor imaging (DTI) enables us to detect the characterization of WM microstructure *in vivo* (Le Bihan, 2003). We employed probabilistic tractography (Behrens et al., 2007) to reconstruct the fiber tracts that seeded from the left FG to the other regions and examined the inter-group differences in the fiber tracts pathway between left FG and other brain areas (Hu et al., 2011).

## Materials and methods

### Subjects

In the present study, 38 healthy volunteers attended the present experiment: an abacus-trained group (*n* = 19; 9 boys; mean age = 10.37 years; standard deviation (SD) = 0.50 years) and a control group (*n* = 19; 9 boys; mean age = 10.14 years; SD = 0.51 years; no specific training was applied in this group). At the beginning of this study, all children were randomly selected to the experimental group for abacus training. After grouping, no children applied to change their group or stop to participate in this program. All participants were from urban families and no participant had any history of neurological or psychiatric disorder. All children studied the same curriculum, apart from the abacus training. The abacus-trained group have received abacus training for over three years and for about 3–4 h per week and the controls received no abacus training either at school or after school. Written informed consent was obtained from each subject and his/her parent prior to MR imaging scanning. This study was approved by Zhejiang University.

### MRI data acquisition

MR images were obtained using a 3-Tesla Philips scanner for each subject. MR sequences included a high-resolution T1-weighted data set with 168 sagittal slices and voxel size of 0.41 × 0.41 × 1 mm (TR/TE = 30/5 ms, FOV = 230 × 230 mm^2^, the acquisition matrix was = 560 × 560).

DTI images were collected on the same scanners using a SENSE coil technology. A DTI pulse sequence with single shot diffusion-weighted echo planar imaging (TR/TE = 5500/78 ms, FOV = 240 × 240 mm^2^, the acquisition matrix was = 288 × 288) was employed sequentially in 15 different directions (*b* = 800 s/mm^2^), together with a non-diffusion-weighted acquisition (*b* = 0 s/mm^2^). We acquired 50 contiguous 3-mm thick slices (no gap) covering the whole brain. DTI images were not acquired from two of the 19 trainees. The duration of the DTI images was 406.4 s. DTI images were not acquired from two of the 19 trainees. The DTI images used in present study were the same data used in our previous research (Hu et al., 2011).

### Imaging analysis

#### Voxel-based morphometry

T1-weighted images were analyzed using VBM8 toolbox in the SPM8 software (Welcome Department of Imaging Neuroscience Group, London, UK). A customized VBM approach was implemented following the combination of the VBM8 toolbox (http://dbm.neuro.uni-jena.de/vbm.html) and the Diffeomorphic Anatomical Registration through Exponentiated Lie algebra toolbox (DARTEL) (Ashburner, 2007). At first processing step, each T1-weighted structural scan was spatially normalized into stereotactic space by coregistering with the standard MNI152 brain template. The “New Segmentation” algorithm from SPM8 was applied to the coregistered image to extract tissue maps corresponding to GM, WM, and cerebrospinal fluid (Ashburner and Friston, 2005; Ashburner, 2009). At second step, the GM, WM and CSF segments were inputted into DARTEL in order to create a customized template. We then normalized each subject's GM segments to this custom template. In this processing we obtained the individual deformation fields. These individual tissue deformations were then used to warp and modulate each participant's GM segments for non-linear effects so that further analyses did not have to account for differences in head size. This is useful for VBM analysis and allows comparing the absolute amount of tissue corrected for individual brain sizes. The modulated GM was written with an isotropic voxel resolution of 1.5 mm. Finally, images were smoothed with a Gaussian kernel of 8 mm (FWHM).

Voxel-wise comparisons of GM volume were performed between the abacus-trained children and the controls using two-sample *t*-tests based on the general linear model. Additionally, we used the left FG masks from the automated anatomical labeling (AAL) template as an explicit mask to restrict the results in left FG (Tzourio-Mazoyer et al., 2002). Age and gender were added as additional covariates in all of these analyses above, which mean that all effect that can be explained by age and sex were removed from the data. The findings were considered significant at a voxel level of *p* < 0.01, FDR corrected for multiple comparisons.

To further investigate the training-related structural changes in these regions in greater detail, we performed a region-of-interest (ROI) analysis. The GM ROI was defined by thresholding at a *p* < 0.01 (FDR corrected) level of significance resulting from the VBM contrast. The sum of the voxel GM volumes inside this ROI was calculated for each subject.

#### DTI: probabilistic tractography

The processing of DTI data was conducted by using the FMRIB Software Library (FSL v4.1.9) (www.fmrib.ox.ac.uk/fsl). Standard processing steps were used, as described in detail previously (Smith et al., 2004). First, due to eddy currents and head motion, the raw 4D datasets were corrected for distortions between volumes by using an affine registration to the first *b* = 0 volume by means of FMRIB's Linear Image Registration Tool (FLIRT: http://www.fmrib.ox.ac.uk/fsl) (Jenkinson and Smith, 2001). Next, non-brain tissue and background noise were removed from *b* = 0 image using the Brain Extraction Tool (BET v2.1) (Smith, 2002). After these steps, FMRIB's Diffusion Toolbox (FDT v2.0) was used to fit the diffusion tensor and calculate the FA, eigenvector and eigenvalue maps. To determine group-level differences in FA between the abacus-trained children and the controls, tract-based spatial statistics for FA images was carried out using package TBSS [part of FSL (Smith et al., 2006)]. For the details of the TBSS results, see our previous work (Hu et al., 2011) and part of results are going to be used in the present study.

Following this, fiber tracking was performed using a probabilistic tractography algorithm implemented in FSL (Probtrackx) and based on Bayesian estimation of diffusion parameters (Bedpostx), following the method previously described by Behrens et al. (2003, 2007). Fiber tracking was initiated from all voxels within the seed masks in the diffusion space to generate 5000 streamline samples, with a step length of 0.5 mm, a curvature threshold of 0.2, and a maximum number of steps of 2000. The seed mask was located in the left FG, defining from the significant groups differences in the structure VBM analysis. This seek mask was then linearly transformed into each subject's native space, where probtrackx was ran.

After all the tracts had been calculated for each subject, we set a 1% threshold (out of the 5000 generated from each seed voxel) to reject low-probability voxels and reduce outlier-induced noise. These selected pathways from each subject were then binarized, transformed to MNI 152 brain standard space, and summed across the subjects to produce group probability map. These group probabilistic maps were set at a threshold that only allowed the paths present in at least of one-third of subjects to be displayed. The John Hopkins University (JHU) white matter tractography atlas was used for tract labeling (Mori et al., 2008). Then the differences in tractography distribution were found between both groups. Subtraction process was used between the group probabilistic maps from the trained group and the controls. The subtraction results were binarized as tractography mask, which was applied on the original FA image. The tractography mask was identical between the trainee and control groups. The mean FA from voxels within this tractography mask were calculated for each subject and exported to SPSS. Because we hypothesize that abacus training would result in neuronal change, we used two-sample *t*-tests to determine significant differences in FA values between the two groups. To seek further neural mechanism of the structural changes in left FG, we assessed the relationship between the GM volume in the left FG and the mean FA value in the tractography mask.

## Results

### Gray matter volume

**Figure 1:**
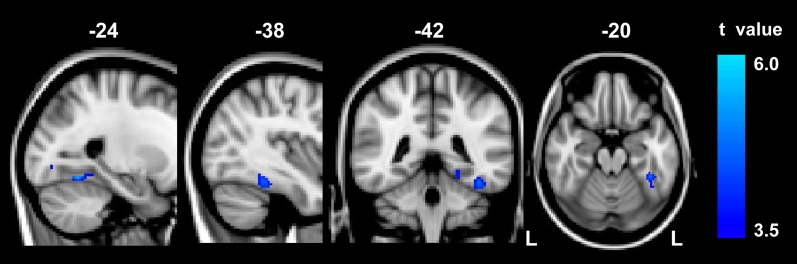
**The result of between-group VBM comparison in the left FG**. Decreased gray matter volume was detected in the abacus-trained group relative to the controls (*p* < 0.01, FDR corrected). The bar to the right indicates the *t* score level. L, left.

Compared to the controls, abacus-trained children showed significantly decreased GM volume in the left FG (Figure 1). The voxels of peak differences in GM volume were at (*x, y, z*) −39, −40, −20 (*t* score = 4.55) and −24, −58, −14 (*t* score = 5.06). The clusters showing significantly group difference were extracted as GM ROI. The sum of the voxel GM volumes inside this ROI was calculated for each subject.

### Probabilistic tractography

**Figure 2:**
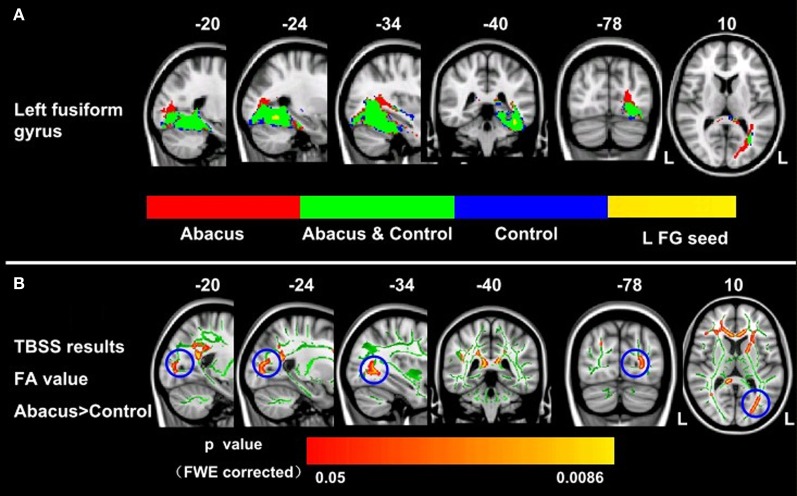
**Group probabilistic tractography pathways connecting left FG for each group. (A)** Average fiber tracts connecting the left FG in abacus-trained group (red) and control group (blue). The probability maps overlapped onto a T1-weighted image (axial, coronal, and sagittal). The green areas indicate the overlap fiber tracts pathways of both groups. L, left. **(B)** White matter structures showed significant enhancement of FA in the abacus-trained group using TBSS method (Hu et al., 2011). The statistical map was overlapped onto the mean FA skeleton (green) and MNI152 template (gray-scale). The FA in left occipitotemporal conjunction area, right premotor projection and corpus callosum were enhanced significantly. The location of left occipitotemporal conjunction area was marked by blue circle. L, left.

**Figure 3:**
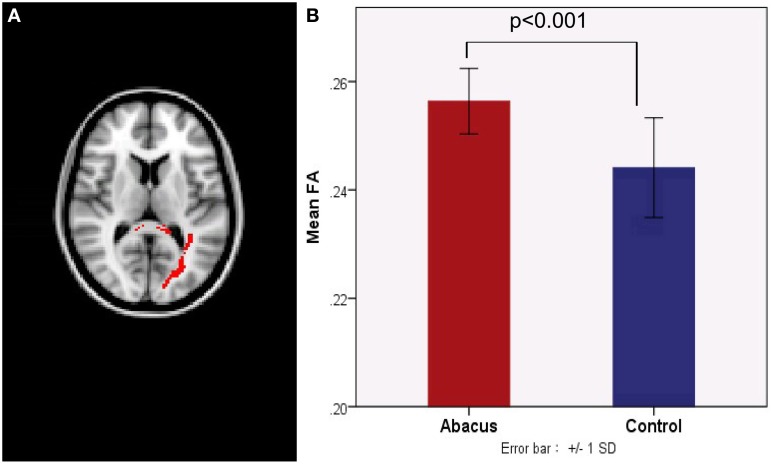
**(A)** A new tract projection pathway, such as left forceps major pathway, was formed from left FG to other brain regions in the abacus-trained group. This tract pathway was extracted as white matter mask. **(B)** Mean fractional anisotropy within this white matter mask was significantly increased in children with abacus training. SD, standard deviation.

Probabilistic tractography was used to examine the probability distribution of fiber pathways from the voxels in the seed mask. Two WM atlases within FSL (TCBM-DTI-81 parcellation map and JHU WM tractography atlas) (Mori et al., 2008) were used to determine the location of the tract result. The primary finding was that the fiber pathways connecting left FG included the sagittal stratum, thalamus radiation and occipitotemporal junction. Based on the JHU WM probabilistic tractography atlas, WM tracts included inferior fronto-occipital fasciculus, and inferior longitudinal fasciculus in all subjects. The probabilistic maps which demonstrated the possible fiber pathways connecting left FG were shown in Figure 2A. In line with our hypothesis, there was a different projection pathway between the abacus-trained group and the controls. Connectivity mapping identified left forceps major as a key pathway connecting left FG with other brain areas in the trained group but not in the controls (Figure 2A). The location of this fiber tract was similar with our previous finding that the FA in left occipitotemporal conjunction area, right premotor projection and corpus callosum were enhanced significantly (Figure 2B) (Hu et al., 2011). Furthermore, the fiber pathway which showed difference between the groups was extracted as tractography mask (Figure 3A). We measured WM microstructure indexed by FA within this tractography mask, where abacus-trained children demonstrated higher mean FA values (abacus-trained group FA = 0.256, control group FA = 0.244; *t* = 4.66, *p* = 0.000; Figure 3B).

**Figure 4:**
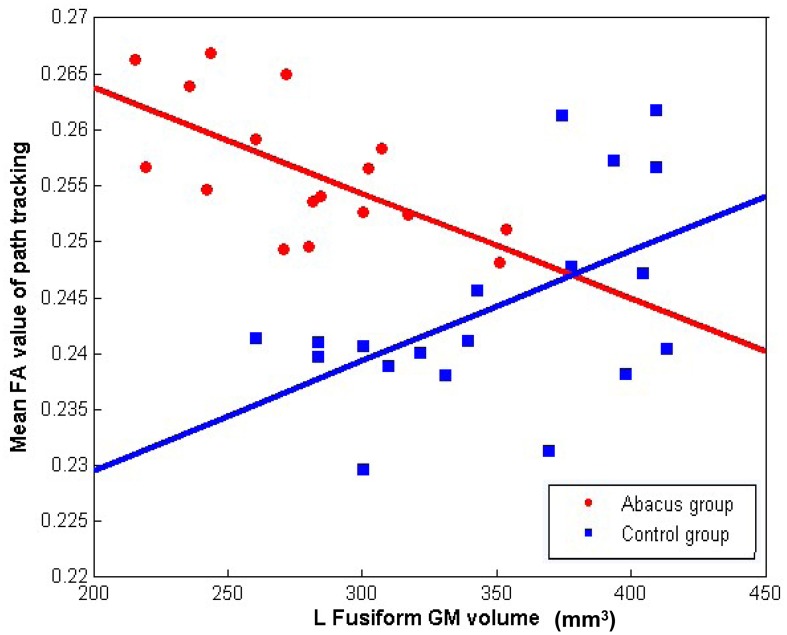
**The correlation between the mean FA value of left forceps major tracts and the GM volume in left FG**. Mean FA values extracted from the tracts showing group difference of connectivity distribution and plotted against the GM volume in left FG showing group difference of structure. Negative correlation was found in the abacus-trained group (red, *r* = −0.64, *p* = 0.006) but positive correlation was found in the controls (blue, *r* = 0.53, *p* = 0.019).

Further analyses were performed to examine the underlying neural mechanism of the structural plasticity in the left FG. Correlation results revealed that GM volume in left FG and the mean FA value in the tractography mask had a significantly negative correlation in the trained group (*r* = −0.64, *p* = 0.006) but a significantly positive correlation in the controls (*r* = 0.53, *p* = 0.019), as shown in Figure 4.

## Discussion

In this study, we demonstrated that long-term abacus training induced structural changes in children's brain. First, VBM analysis on the T1-weighted images identified that GM in the trained children had a significantly smaller volume in left FG than the controls. Second, as revealed by probabilistic tractography, connectivity mapping identified left forceps major as a key pathway connecting left FG with other brain areas in the trained group but not in the controls. Mean FA value within the left forceps major tracts was enhanced in the trained group. Third, significantly negative correlation was found between the GM volume in left FG and the mean FA value of left forceps major fiber tracts only in the trained group.

Our findings indicated that long-term abacus training could induce the structural reduction of left FG and increase the intensity of left forceps major fiber tracts. Moreover, the negative relationship between GM volume and mean FA supports the theory of inverse effect (Golestani et al., 2002; Draganski et al., 2006; May, 2011). Our findings demonstrated that the structural reduction of left FG and the increased fiber integrity of left forceps major fiber tracts possibly attributed to the long-term abacus training.

### The macro-structural measurement of the GM

Evidences from neuroplasticity studies suggested that long-term skill acquisition and experience could cause functional and structural reorganization of the brain (Chen et al., 2006; Bezzola et al., 2011; May, 2011; Ansari, 2012). Mental abacus is a system for performing rapid and precise arithmetic operations by manipulating a mental representation of an abacus (Frank and Barner, 2012). These works demonstrated the important role of mental imagery in abacus mental calculation. In the present study, VBM analysis on T1-weighted data revealed reduced GM volume in the left FG, possibly due to its frequent engagement in visuospatial processing in abacus-based mental calculations. Long-term abacus training might help trainee transform the visual resources into the mental abacus. Previous studies have shown the parietal cortex functionally interconnects with the FG, which is a part of the ventral visual pathway (Büchel et al., 1999). Visual information might be transformed into a super-modal form of abacus beads through the left FG and then transmitted to the frontoparietal network for mental calculations. During the numeral mental-operation task, a number size effect was found in the left FG of abacus-trained group (Hanakawa et al., 2003), which provided the functional neuroimaging evidence for our present study.

For anatomical changes of human brain in response to training, previous studies suggested that the change in cortical GM is the result of a complex array of morphological changes including simple changes in cell size, growth or atrophy of neurons or glia (Duerden and Laverdure-Dupont, 2008; Anderson, 2011). The new connections were formed by dendritic spine growth or the strength of existing connections was changed (Holtmaat et al., 2006). Although most of the brain imaging studies on learning and training report the selective enlargement of the brain regions responsible for the specific skill being studied (Anderson, 2011; Bezzola et al., 2011), trainings leading to decrease in GM were reported recently (Berkowitz and Ansari, 2010; Takeuchi et al., 2011; Duan et al., 2012). Our VBM results on the T1-weighted images also found a training-dependent decrease of GM, resulting possibly form the neural pruning in the left FG induced by long-term abacus training. Redundant or unused synapses were removed for the neural pruning selection. After pruning, it is possible that fewer synapses are required to do same amount of work, and thus the efficiency of local neuronal connections was enhanced (Blakemore, 2012).

### The micro-structural measurement of the WM

Further evidence for structural plasticity comes from the probabilistic tractography results. Connectivity distribution mapping provided detailed information about the major tracts that run through left FG. Group differences were observed in the tract distribution mapping connecting the left FG. The abacus-trained group showed a significantly stronger connection than the controls in the left forceps major pathway. The abacus-trained group also showed a substantial increase in the integrity of the left forceps major. Our tractography analyses perfectly reproduced the results of our previous study that the FA value in the WM tract trajectory of left forceps major increased significantly in the abacus-trained group (Hu et al., 2011). As a subset of the corpus callosum, the forceps major links the bilateral occipital lobes through the splenium. The intensity of the fiber paths actually represents the changes in the WM micro-structure. Along the trajectory of the left forceps major, significant increase in FA possibly reflected some changes in some aspects of connectivity (Jones et al., 2013). Thus, our tractography results indicated that long-term abacus training possibly changed the structural connectivity between the occipital and left FG.

Combining with our VBM results on T1-weighted images, we found a decrease of GM in the left FG but a highly significant increase of WM in the adjacent region. This phenomenon can be explain by the theory of inverse effect (Golestani et al., 2002; Draganski et al., 2006). A reduction in GM volume would prompt an inverse effect in WM in the adjacent region. Unused synapses were removed and the remaining synapses were more efficient for information processing. A brain imaging study on chess experts also found this kind of neural pruning (Duan et al., 2012). They found the GM volume decreased in caudate but the functional connections increased between the caudate and the default mode network. Chess experts rely on a rich network of chess patterns stored in the brain. Relevant information was accessed quickly and the automatic process was formed in chess playing. In another study (Draganski et al., 2006), a decrease of GM induced by extensive learning on adults was found in the occipital parietal lobe, which was accompanied by a highly significant increase of WM in an adjacent region. In our study, FA increased in the WM regions but adjoining areas showed a decrease in the GM volume. Our structural results were in accordance with these previous studies (Draganski et al., 2006; Duan et al., 2012) and possibly suggest that enhanced information processing capacity was established through local structural plasticity.

Moreover, the micro-structural plasticity is left lateralized in the training group. Previous functional neuroimaging studies have observed that the left FG was involved in abacus mental calculation (Hanakawa et al., 2003; Chen et al., 2006). This possibly indicates a preferential role of the left FG in abacus-based mental calculations.

### The relationship between the GM volume and the WM FA value

As demonstrated in age-related alteration in brain structure, there is a high correlation between volume changes and FA changes (Hugenschmidt et al., 2008). Age-related WM changes were intimately related to GM changes. Interestingly, in the present study, we demonstrated a strong negative relationship between the GM volume in the left FG and the mean FA value in left forceps major in abacus-trained group but a positive relationship in controls. Both WM integrity changes and neural pruning occur in the abacus-trained children. WM integrity changed in a voxel, leading to increased FA (Stikov et al., 2011), whereas unused synapses were eliminated, leading to decreased GM volume (Draganski et al., 2006). The inverse effect phenomenon has been reported in some neuroimaging studies (Golestani et al., 2002; Draganski et al., 2006), the present study extended this finding by further examining the relationship between GM volume and the WM FA value. This relationship highlighted the phenomenon that a reduction in GM volume would prompt an inverse effect in adjacent WM. Beyond this, our findings indicated that long-term abacus training could significantly alter the brain both at the macro- and micro-structural level. In the control group, a significant positive correlation between GM volume and WM FA was found. This result can be explain by pediatric brain development. Research using MRI to acquire structural images from developing children and adolescents demonstrate inverted U shaped trajectories of GM volumes with peak sizes occurring at different regions (Giedd and Rapoport, 2010; Blakemore, 2012). For example the volume of GM in the frontal lobe and parietal lobe increased during late children to a peak at around 12 years (Blakemore, 2012). A longitudinal study of 103 participants from 5 to 32 years showed non-linear development trajectories for FA (Lebel and Beaulieu, 2011). Both the GM volume and the FA value showed increased during late childhood and early adolescence. A positive correlation may be present between the GM volume and the WM FA in the brain before 12 years. In the present study, all participants were about 10 years old. The positive correlation between GM volume and WM FA in the control group may be the earlier maturation result.

The present study had some limitations. First, this is a cross-sectional study which cannot completely eliminate the distraction on the structural difference. A longitudinal design is a better choice to provide elaborate understanding of brain plasticity. Second, brain structural changes can precede functional changes, or alternatively (Blakemore, 2012). Future research should combine the structural and functional methods to identify the role of left FG in the abacus mental calculation.

## Conclusions

In conclusion, we found that abacus-trained group had significantly anatomical changes in the left FG. The volume of left FG reduced significantly and a new tract projection, such as left forceps major pathway, was formed from left FG to other brain regions in the abacus-trained group. In this new tract projection, the fiber integrity was enhanced significantly in the abacus-trained group. It is reasonable to say that abacus training-dependent structural changes occur both at a macro- and at a micro-structural level. The negative correlation between GM volume and the WM FA value can be explained by the theory of invert effect. These results provide insights into the neural mechanism of structural plasticity induced by abacus training. Furthermore, there is a fact that there conclusions may apply to the children only, because this study was conducted on the children. Brain maturation is a complex process, during which brain functions and neurostructures could be modulated by training. The onset of the training is a crucial factor in domain-specific brain modulations. These GM and WM structural plasticity in the present study may not be seen in adult following the abacus training.

### Conflict of interest statement

The authors declare that the research was conducted in the absence of any commercial or financial relationships that could be construed as a potential conflict of interest.
